# Prognostic Role of MicroRNA-200c-141 Cluster in Various Human Solid Malignant Neoplasms

**DOI:** 10.1155/2015/935626

**Published:** 2015-10-18

**Authors:** Xiao-yang Li, Hui Li, Jie Bu, Liang Xiong, Hong-bin Guo, Li-hong Liu, Tao Xiao

**Affiliations:** Department of Orthopedics, The Second Xiangya Hospital of Central South University, Changsha 410011, China

## Abstract

The miR-200 family has emerged recently as a noticeable marker for predicting cancer prognosis and tumor progression. We aimed to review the evidence of miR-200c-141 genomic cluster as prognostic biomarkers in cancers. The results suggested that high level of miR-200c had no significant impact on OS (HR = 1.14 [0.77–1.69], *P* = 0.501) and DFS/PFS (HR = 0.72 [0.45–1.14], *P* = 0.161). Stratified analyses revealed that high miR-200c expression was significantly related to poor OS in serum/plasma (HR = 2.12 [1.62–2.77], *P* = 0.000) but not in tissues (HR = 0.89 [0.58–1.37], *P* = 0.599). High miR-200c expression was significantly associated with favorable DFS/PFS in tissues (HR = 0.56 [0.43–0.73], *P* = 0.000) but worse DFS/PFS in serum/plasma (HR = 1.90 [1.08–3.36], *P* = 0.027). For miR-141, we found that high miR-141 expression predicted no significant impact on OS (HR = 1.18 [0.74–1.88], *P* = 0.482) but poor DFS/PFS (HR = 1.11 [1.04–1.20], *P* = 0.003). Similarly, subgroup analyses showed that high miR-141 expression predicted poor OS in serum/plasma (HR = 4.34 [2.30–8.21], *P* = 0.000) but not in tissues (HR = 1.00 [0.92–1.09], *P* = 0.093). High miR-141 expression was significantly associated with worse DFS/PFS in tissues (HR = 1.12 [1.04–1.20], *P* = 0.002) but not in serum/plasma (HR = 0.90 [0.44–1.83], *P* = 0.771). Our findings indicated that, compared to their tissue counterparts, the expression level of miR-200c and miR-141 in peripheral blood may be more effective for monitoring cancer prognosis. High miR-141 expression was better at predicting tumor progression than survival for malignant tumors.

## 1. Introduction

Cancer is a global major public health issue [1, 2]. It accounts for one of the leading causes of mortality prevalent in most regions worldwide [3]. According to a GLOBOCAN report, the global burden of cancer continued to increase largely. in 2012, cancer death cases were estimated up to 8.2 million, and most of them occurred in developing countries [4]. Although multidisciplinary treatments including chemotherapy, radiotherapy, and surgery have remarkably improved the survival of cancer in the last decades, local and metastatic relapses have been consistently shown to dramatically reduce survival. Thus advanced biomarkers are necessary for proper prediction of cancer prognosis [5].

MicroRNAs (miRNAs) are a class of evolutionarily conserved endogenous small noncoding molecules. These single-stranded 18–25 nucleotides long RNAs could sequence-specifically regulate gene expression and various biological processes [6, 7]. Since the initial discovery identified miRNAs in 1993, emerging evidences from clinical researches have indicated that miRNAs are crucial in cellular differentiation, growth, stress response, cell death, and other fundamental cellular processes, and their involvement in malignant neoplasms has been confirmed [8]. Recently, miRNA expression profiling has revealed that certain miRNAs were implicated in tumorigenesis, tumor progression, and clinicopathological features of cancers [9–11]. Therefore, miRNAs could be promising prognostic biomarker candidates in various human cancers [12–14].

The miRNA-200 family consisting of five highly homologous members (miR-200a, miR-200b, miR-200c, miR-429, and miR-141) can be separated into two gene clusters based on the fact that they are expressed from two distinct polycistronic transcripts; the miR-200b/a/429 cluster is located on chromosome 1p36, and the miR-200c/141 cluster is located on chromosome 12p13 [14, 15]. The miR-200 family has emerged recently as a significant marker, as well as a pivotal regulator of the epithelial-to-mesenchymal transition (EMT) in a variety of cancers [14, 16–18]. Increasing evidence demonstrated that the cluster of miR-200c-141 seems to have a dual role in patient prognosis. However, consensus has not been reached to the reliability of miR-200c and miR-141 as prognostic biomarkers in tumors [19–21]. Hence, the prognostic relevance of miR-200c and miR-141 expression in cancer remains controversial. Considering the weakness of individual study, it is essential to conduct a meta-analysis to address the inconsistence by systematically summarizing available findings.

Therefore, in this study, we performed a comprehensive meta-analysis to clarify the prognostic value of miR-200c and miR-141 expression in human cancers.

## 2. Materials and Methods

This meta-analysis was conducted in accordance with the standard guidelines of Preferred Reporting Items for Systematic Reviews and Meta-Analyses (PRISMA) 2009 Checklist (http://www.prismastatement.org/statement.htm) and Meta-Analysis of Observational Studies in Epidemiology group (MOOSE) [43].

### 2.1. Search Strategy

To obtain relevant literatures for this meta-analysis, we systematically and carefully searched the online PubMed (http://www.ncbi.nlm.nih.gov/pubmed), Embase (http://www.embase.com/home), and Web of Science (http://wokinfo.com/) up to September 30, 2014. No language or other restrictions were made. The following three sets of key words and their combination search terms were simultaneously applied, namely, “miR-200c OR miR-141,” “cancer OR carcinoma OR tumor OR malignant neoplasm,” and “survival OR prognosis OR outcome.” All the searching records were reviewed by going through the titles and abstracts. The duplications were removed directly.

A manual search was conducted to identify additional prospective studies by using cited references from relevant original articles, reviews, and editorials on this topic. If more than one miR-200 family member or cancer type was reported in one study, each was extracted separately. The most complete study was included in our analysis when there was more than one study containing overlapping data from the same authors. Requests were emailed to the authors when supplementary information and essential data are needed.

Information of the eligible reports, such as titles, abstracts, full texts, and reference lists were independently and carefully identified from all of the publications in triplicate by two reviewers (Xiao-yang Li and Xiong). Bu and Guo double checked these extracted articles for a second time. Disagreements were resolved by discussion among these reviewers (Xiao-yang Li, Xiong, Bu, and Guo) and consultation with senior reviewers (Liu and Hui Li).

### 2.2. Inclusion Criteria and Exclusion Criteria

Studies were considered eligible according to the following criteria: (i) any type of human solid tumor was studied; (ii) the expression of miR-200c or miR-141 in tumor tissue or blood sample was measured; (iii) the associations between miR-200c or miR-141 expression and survival outcome were investigated; and (iv) sufficient data was provided to estimate hazard ratios (HRs) and corresponding 95% confidence intervals (95% CIs) for survival rates.

Articles were excluded if they met the following criteria: (i) hematological malignancies and autoimmune disorders; (ii) studies analyzing a set of miRNAs altogether other than a separated one; (iii) reviews, case reports, comments, economic analyses, conference abstracts, animal studies, and laboratory studies; (iv) lack of crucial information about survival outcome or not being able to estimate HR and 95% CI by the available data.

### 2.3. Quality Assessment

Quality assessment for all the included studies was systematically performed independently by three investigators (Xiao-yang Li, Bu, and Hui Li), based on the critical guidelines of the Dutch Cochrane Centre proposed by MOOSE for prognostic meta-analysis [43]. The key points of the review checklist included the following: (i) clear description of study population and origin of country, (ii) clear definition of type of carcinoma, (iii) clear explanation of study design, (iv) clear description of outcome assessment, (v) clear report of miR-200 family measurement, (vi) clear definition of cut-off of miR-200 family, and (vii) sufficient follow-up period. We excluded the studies without specifying any aspect concerning the above so as not to compromise the quality of the meta-analysis.

### 2.4. Data Extraction and Conversion

The two investigators (Xiao-yang Li and Bu) independently extracted relevant information in standardized data collection forms to rule out any discrepancy. The following characteristics of the individual eligible research articles were collected: the first author's name, year of publication, origin of population, selection of number of cases, cancer type, and sample source, the member of miR-200 family, validation methods, cut-off values, survival results, and prognosis.

HRs with their 95% CIs were extracted according to the following three methods [44]. Only reported univariate analysis results for survival in eligible studies were considered for the aggregation of the survival data. In most instances, the reported HRs with their 95% CIs and *P* values were directly derived from the original publications or the corresponding E-mails from the authors, with an HR of >1 being associated with elevated risk of mortality or recurrence, which is the most accurate method. In absence of HRs and 95% CIs, the total numbers of observed deaths/cancer recurrences and the numbers of samples in each group or the valuable data provided by the authors were extracted to calculate HRs. If only Kaplan-Meier curves are available, data were extracted from the graphical survival plots to estimate the HRs following the previously described method [44, 45]. If needed, we sought original data directly from the authors of the relevant studies. All the results extracted according to the above three methods were compared, and disagreements were discussed among all the authors to resolve with consensus.

### 2.5. Statistical Analysis

The survival outcome of cancer associated with miR-200c or miR-141 expression was estimated by using the hazard ratio (HR) and their associated 95% confidence intervals (95% CI) for each study. HRs with 95% CIs were used to combine the pooled data. Heterogeneity of combined HRs was assessed by Cochran's *Q* test and Higgin's *I* ^2^ statistic [46, 47]. Heterogeneity was considered statistically significant as *P* < 0.05 or *I* ^2^ > 50%. Pooled HR was calculated using a fixed-effects model or random-effects model to evaluate the relationship between miR-200c or miR-141 expression and survival rate. A fixed-effects model (Mantel-Haenszel test) was applied in the absence of between-study heterogeneity (*P* ≥ 0.05 or *I* ^2^ ≤ 50%) [48], while the random-effects model (Der Simonian and Laird method) was applied if significant heterogeneity was observed (*P* < 0.05 or *I* ^2^ > 50%) [49].

In order to seek possible explanations for heterogeneity, stratified analyses were performed by classifying studies into subgroups of sample source, ethnicity, and main cancer type. Analyses were conducted for all studies and differences between the subgroups which were assessed using methods described by Julian and Higgins [50]. To validate the credibility of outcomes in this meta-analysis, analysis of sensitivity was performed to evaluate the stability of the results; namely, each single study in the meta-analysis was omitted at a time to reflect the influence of the individual data set on the results.

The Begg's funnel plot and Egger's bias indicator test were used to evaluate the potential publication bias among the included studies [51, 52]. *P* < 0.05 in all the two-sided statistical tests was regarded as significant. No corrections were made for multiple comparisons. All analyses were conducted using the STATA package version 12.0 (Stata Corporation, College Station, Texas, USA).

## 3. Results

### 3.1. Eligible Studies

**Figure 1:**
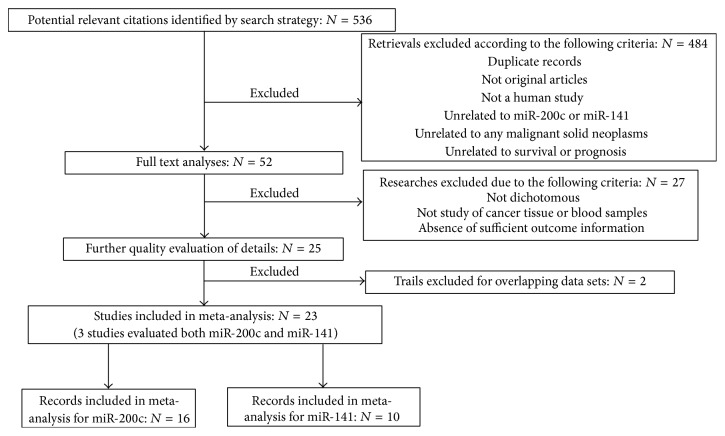
Flow diagram of the identification and selection of studies.

A total of 536 studies were identified after searching in PubMed, Embase, and Web of Science for publications on miR-200c and miR-141 expression associated with cancer prognosis. The titles, publication types, and abstracts were initially evaluated and the full texts were further reviewed. Finally, 23 studies that met the inclusion criteria were considered qualified for the present meta-analysis [19, 20, 22–42]. Of the eligible studies, 13 studies separately evaluated miR-200c [20, 22–25, 27–30, 33–36], 7 studies separately evaluated miR-141 [19, 37–42], and 3 studies simultaneously evaluated miR-200c and miR-141 [26, 31, 32].  Figure 1 showed the flow diagram of candidate study selection in our study.

### 3.2. Characteristics of Included Studies

We collected the essential data from the enrolled 23 studies which were conducted between 2010 and 2014. A total of 2489 participants from different territories involving the United States, Spain, Japan, China, South Korea, Italy, Australia, Germany, and Portugal were included in this meta-analysis. The sample size of the included study ranged from 34 to 212 patients. A wide range of human solid malignant neoplasms were investigated in these eligible 23 studies including colorectal cancer, esophageal cancer, gastric cancer, pancreatic cancer, ovarian cancer, endometrial cancer, lung cancer, bladder cancer, prostate cancer, renal cancer, and hepatocellular carcinoma.

The expression of miR-200c and miR-141 was measured in collected cancerous tissues in the majority of studies except seven targeted in circulation samples [23–25, 27, 29, 37, 39], including one researched in cancerous tissues and blood samples meanwhile [29]. Quantitative real-time polymerase chain reaction (qRT-PCR) assay was widely applied to detect the expression level of miR-200c and miR-141 except two studies which used in situ hybridization (ISH) [30, 38]. The expression levels of miR-200c and miR-141 were dichotomized in all these 23 studies, but the cut-off value was different, with median, mean, and defined level.

Included studies in this meta-analysis referred to evaluating miR-200c and miR-141 expression for overall survival (OS), disease-free survival (DFS), progression-free survival (PFS), and disease-specific survival (DSS). For quantitative analyses, the pooled HRs along with their 95% CIs of all available trials were grouped into OS and DFS/PFS (including DSS for miR-141). As to the data extraction methods, the HRs and 95% CIs which were reported by univariate regression were directly used for 15 studies, and the other HRs and 95% CIs were calculated based on available numerical data or Kaplan-Meier curves for the remaining 8 studies. The main features of these 23 studies were summarized in Table 1 for miR-200c and Table 2 for miR-141.

### 3.3. Overall Survival (OS) Associated with miR-200c Expression

**Figure 2:**
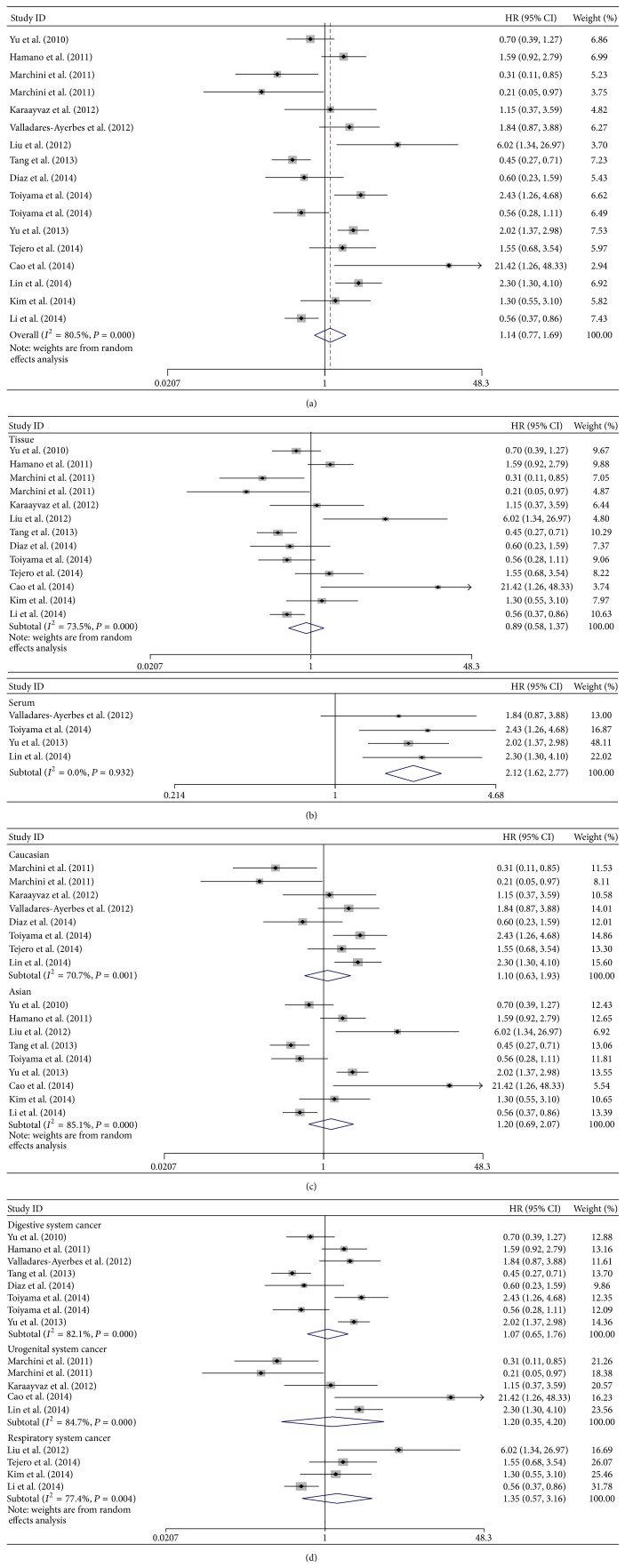
Forest plots of merged analyses for overall survival (OS) associated with miR-200c expression. (a) Forest plot to assess the overall effect; (b) Forest plots for the subgroup analysis in different detected samples; (c) Forest plots of ethnic effect; (d) Forest plots for the subgroup analysis in different malignant diseases.

For studies evaluating OS for miR-200c, a random-effects model was applied to calculate the pooled HR and its 95% CI because of the high significant heterogeneity which had been found in the 17 cohorts (*I* ^2^ = 80.5%, *P* = 0.000). The result showed that high level of miR-200c may predict poorer OS, with the pooled HR being 1.14 (95% CI: 0.77–1.69). However, the effect did not reach the level of statistical significance (*P* = 0.501) (Figure 2(a)).

Stratified analyses were performed by classifying studies into subgroups of sample source, dominant ethnicity, and malignant diseases. In the subgroup analysis of sample source, no significant association between the high level of miR-200c in tissue and overall survival was found (pooled HR = 0.89; 95% CI: 0.58–1.37; *P* = 0.599) by a random-effects model (*I* ^2^ = 73.5%, *P* = 0.000). However, significant effect was observed between the high level of miR-200c in serum/plasma and poorer OS (pooled HR = 2.12; 95% CI = 1.62–2.77; *P* = 0.000) by a fixed-effects model (*I* ^2^ = 0.0%, *P* = 0.932) (Figure 2(b)). When stratified by the dominant ethnicity, we did not find a significantly worse OS (random-effects model: pooled HR = 1.10; 95% CI: 0.63–1.93; *P* = 0.734) in Caucasians. Similarly, the result (random-effects model: pooled HR = 1.20; 95% CI: 0.69–2.07; *P* = 0.515) showed that the association between high level of miR-200c and poor OS was not significant in Asians (Figure 2(c)). In subtotal analyses of main malignant type, no significant results were observed in digestive system cancers (random-effects model: pooled HR = 1.07; 95% CI: 0.65–1.76; *P* = 0.793), urogenital system cancers (random-effects model: pooled HR = 1.20; 95% CI: 0.35–4.20; *P* = 0.770), and respiratory system cancers subgroup (random-effects model: pooled HR = 1.35; 95% CI: 0.57–3.16; *P* = 0.496) (Figure 2(d)).

### 3.4. Tumor Progression (DFS/PFS) Associated with miR-200c Expression

**Figure 3:**
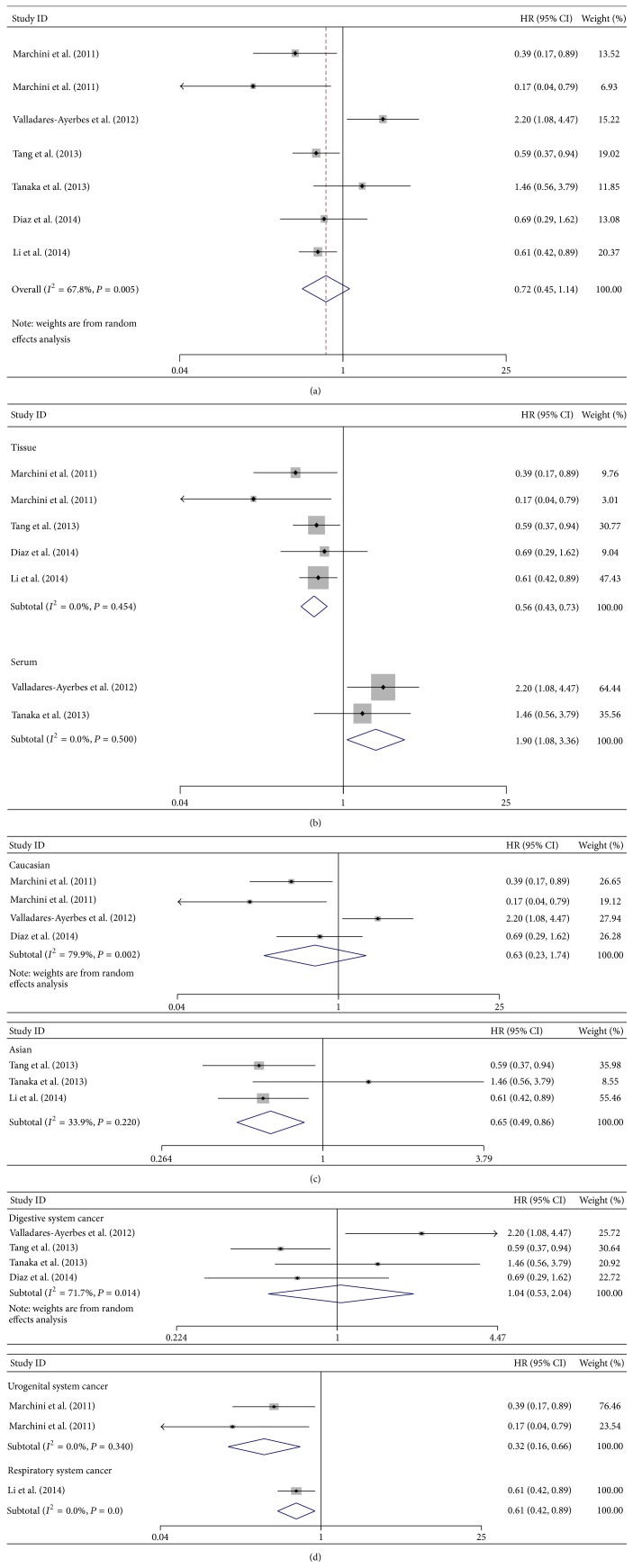
Forest plots of merged analyses for disease-free survival (DFS) and progression-free survival (PFS) associated with miR-200c expression. (a) Forest plot to assess the overall effect; (b) Forest plots for the subgroup analysis in different detected samples; (c) Forest plots of ethnic effect; (d) Forest plots for the subgroup analysis in different malignant diseases.

We analyzed tumor progression associated with high miR-200c expression by combining disease recurrence and metastasis. A total of seven studies focused on DFS/PFS (including DFS and PFS) analysis with a significant heterogeneity among them (*I* ^2^ = 67.8%, *P* = 0.005). A random-effects model was applied and no obvious relationship between high level of miR-200c and DFS/PFS was shown (pooled HR = 0.72; 95% CI: 0.45–1.14; *P* = 0.161) (Figure 3(a)).

Similar to OS analyses, we also performed subtotal investigation for DFS/PFS analyses. In subgroup analysis stratified by detected samples, high level of miR-200c in serum/plasma exhibited a significant association with poor DFS/PFS (HR = 1.90; 95% CI: 1.08–3.36; *P* = 0.027) and no heterogeneity was observed (*I* ^2^ = 0.0%, *P* = 0.500). However, the pooled outcome in tissue subgroup surprisingly showed that high miR-200c expression was significantly associated with a favorable DFS/PFS (HR = 0.56; 95% CI: 0.43–0.73; *P* = 0.000) by a fixed-effects model (*I* ^2^ = 0.0%, *P* = 0.454) (Figure 3(b)). When stratified by dominant ethnicity, no significant association was observed in Caucasians (pooled HR = 0.63; 95% CI: 0.23–1.74; *P* = 0.370) by a random-effects model (*I* ^2^ = 79.9%, *P* = 0.002), but high level of miR-200c significantly associated with favorable DFS/PFS (pooled HR = 0.65; 95% CI: 0.49–0.86; *P* = 0.002) in Asians by fixed-effects model (*I* ^2^ = 33.9%, *P* = 0.220) (Figure 3(c)). Finally, the results revealed that high level of miR-200c significantly associated with favorable DFS/PFS in respiratory system cancers (HR = 0.61; 95% CI: 0.42–0.89; *P* = 0.010) and urogenital system cancers (pooled HR = 0.32; 95% CI: 0.16–0.66; *P* = 0.002) by a fixed-effects model (*I* ^2^ = 0.0%, *P* = 0.340). We did not find a significant favorable DFS/PFS (pooled HR = 1.04; 95% CI: 0.53–2.04; *P* = 0.917) in digestive system cancers by a random-effects model (*I* ^2^ = 71.7%, *P* = 0.014) (Figure 3(d)).

### 3.5. Overall Survival (OS) Associated with miR-141 Expression

**Figure 4:**
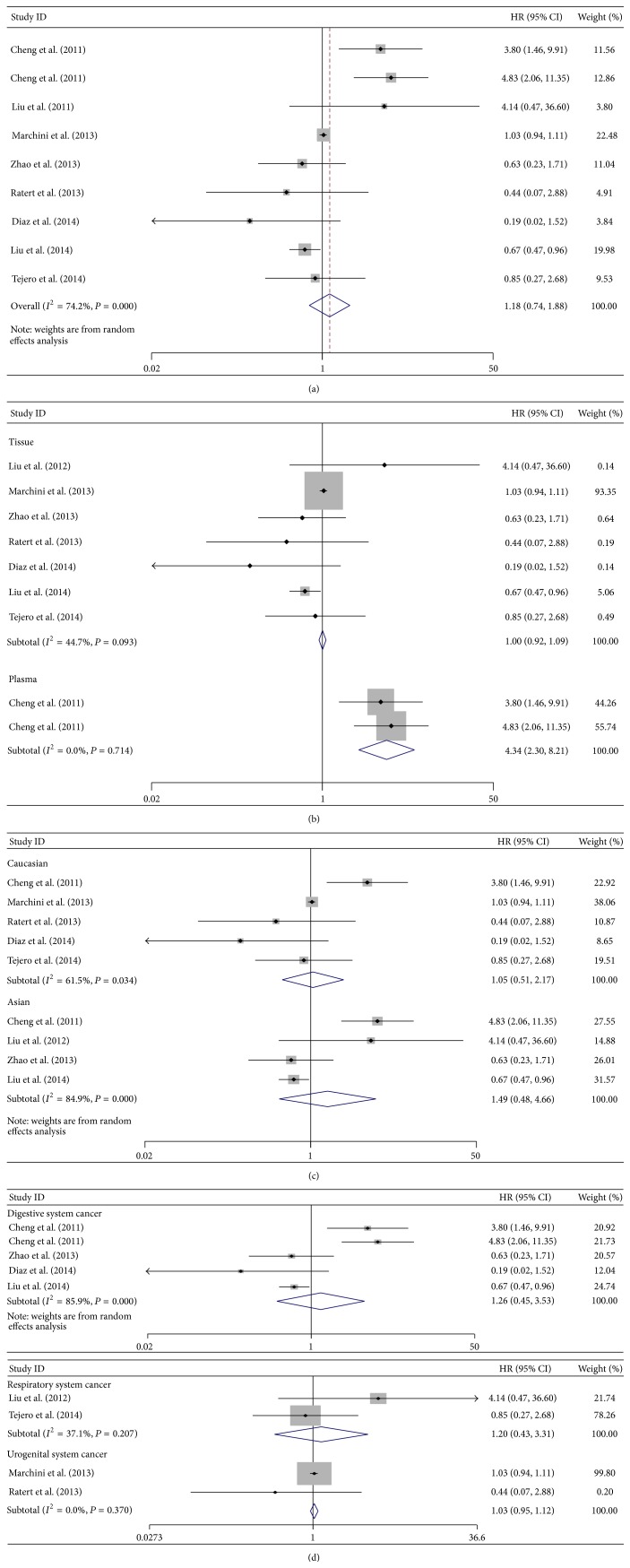
Forest plots of merged analyses for overall survival (OS) associated with miR-141 expression. (a) Forest plot to assess the overall effect; (b) Forest plots for the subgroup analysis in different detected samples; (c) Forest plots of ethnic effect; (d) Forest plots for the subgroup analysis in different malignant diseases.

For the studies evaluating OS for miR-141, a random-effects model was used to calculate the pooled HR with 95% CI due to the significant heterogeneity (*I* ^2^ = 74.2%, *P* = 0.000), and no statistically significant relevance was observed (pooled HR = 1.18; 95% CI: 0.74–1.88; *P* = 0.482) (Figure 4(a)).

Subgroup analyses failed to exhibit a significant association between high level of miR-141 and overall survival in tissue subgroup (pooled HR = 1.00; 95% CI: 0.92–1.09; *P* = 0.967) by a fixed-effects model (*I* ^2^ = 44.7%, *P* = 0.093) but showed that high level of miR-141 in serum/plasma was a significant prediction for poor OS (pooled HR = 4.34; 95% CI: 2.30–8.21; *P* = 0.000) by a fixed-effects model (*I* ^2^ = 0.0%, *P* = 0.714) (Figure 4(b)). When stratified by dominant ethnicity, no significant relevance was observed in both Caucasians (random-effects model: pooled HR = 1.05; 95% CI: 0.51–2.17; *P* = 0.887) and Asians (random-effects model: pooled HR = 1.49; 95% CI: 0.48–4.66; *P* = 0.493) (Figure 4(c)). Moreover, in subtotal analyses of malignant diseases, no significant association was displayed in subgroups of digestive system cancers (pooled HR = 1.26; 95% CI: 0.45–3.53; *P* = 0.667) by a random-effects model (*I* ^2^ = 85.9%, *P* = 0.000), respiratory system cancers (pooled HR = 1.20, 95% CI: 0.43–3.31, *P* = 0.726) by a fixed-effects model (*I* ^2^ = 37.1%, *P* = 0.207), and urogenital system cancers (pooled HR = 1.03; 95% CI: 0.95–1.12; *P* = 0.511) by a fixed-effects model (*I* ^2^ = 0.0%, *P* = 0.370) (Figure 4(d)).

### 3.6. Tumor Progression (DFS/PFS) Associated with miR-141 Expression

**Figure 5:**
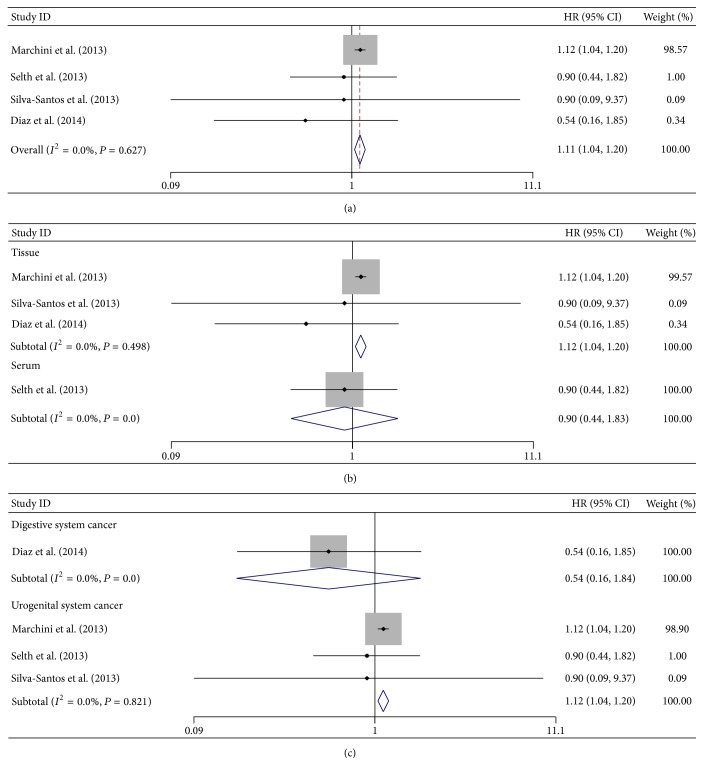
Forest plots of merged analyses for disease-free survival (DFS) and progression-free survival (PFS) associated with miR-141 expression. (a) Forest plot to assess the overall effect; (b) Forest plots for the subgroup analysis in different detected samples; (c) Forest plots for the subgroup analysis in different malignant diseases.

We analyzed tumor progression associated with miR-141 expression by combining disease recurrence, metastasis, and disease death. Meta-analysis of the eligible studies predicted that high level of miR-141 was significantly associated with poor DFS/PFS (pooled HR = 1.11; 95% CI: 1.04–1.20; *P* = 0.003). No significant heterogeneity was observed (*I* ^2^ = 0.0%, *P* = 0.627) and the fixed-effects model was applied (Figure 5(a)).

Further stratified analyses by detected sample type displayed that high level of miR-141 remained to be a worse prognostic marker in tissue subgroup (pooled HR = 1.12; 95% CI: 1.04–1.20; *P* = 0.002) by a fixed-effects model (*I* ^2^ = 0.0%, *P* = 0.498) but failed to show a significant association between miR-141 expression and tumor progression in serum/plasma (HR = 0.90; 95% CI: 0.44–1.83; *P* = 0.771) (Figure 5(b)). Since all the eligible studies focusing on miR-141 expression and tumor progression were carried out with Caucasian cases, subtotal analysis conducted by the ethnicity was not performed. When different malignant diseases were considered, the result revealed that high miR-141 expression in urogenital system cancers was associated with a poor DFS/PFS (pooled HR = 1.12; 95% CI: 1.04–1.20; *P* = 0.002) by the fixed-effects model (*I* ^2^ = 0.0%, *P* = 0.821). However, subgroup analysis in digestive system cancers showed no statistical significance (HR = 0.54; 95% CI: 0.16–1.84; *P* = 0.324) (Figure 5(c)).

### 3.7. Heterogeneity Analysis Results

Heterogeneity was observed among studies evaluating OS for miR-200c (OS for all, *I* ^2^ = 80.5%) and miR-141 (OS for all, *I* ^2^ = 74.2%), as well as studies evaluating DFS/PFS for miR-200c (DFS/PFS for all, *I* ^2^ = 67.8%). Then, we, respectively, assessed the source of heterogeneity comparison by detected sample source, ethnicity, and cancer type. Substantial heterogeneity was discovered in tissue subgroup for miR-200c (OS as endpoint, *I* ^2^ = 73.5%), as well as tissue subgroup for miR-141 (OS as endpoint, *I* ^2^ = 44.7%). The heterogeneity was partly decreased in Caucasians in some subgroup analyses (OS for miR-200c: *I* ^2^ = 70.7%; OS for miR-141: *I* ^2^ = 61.5%). However, there was still significant heterogeneity among Asians in some subgroup analyses (OS for miR-200c: *I* ^2^ = 85.1%; OS for miR-141: *I* ^2^ = 84.9%). In subgroup analyses of tumor type evaluating OS for miR-200c, heterogeneity was seen in digestive system cancer (*I* ^2^ = 82.1%), respiratory system cancer (*I* ^2^ = 77.4%), and urogenital system cancer (*I* ^2^ = 84.7%). The subgroup analyses of tumor type evaluating DFS/PFS for miR-200c showed that the heterogeneity was significant among digestive system cancer (*I* ^2^ = 71.7%), while no heterogeneity was observed in other subgroups. Similar kind of considerations held for the subgroup analyses of tumor type for miR-141, since the heterogeneity of OS for digestive system cancers was obvious (*I* ^2^ = 85.9%), while no significant heterogeneity was viewed in other cancer types.

### 3.8. Publication Bias and Sensitivity Analysis

**Figure 6:**
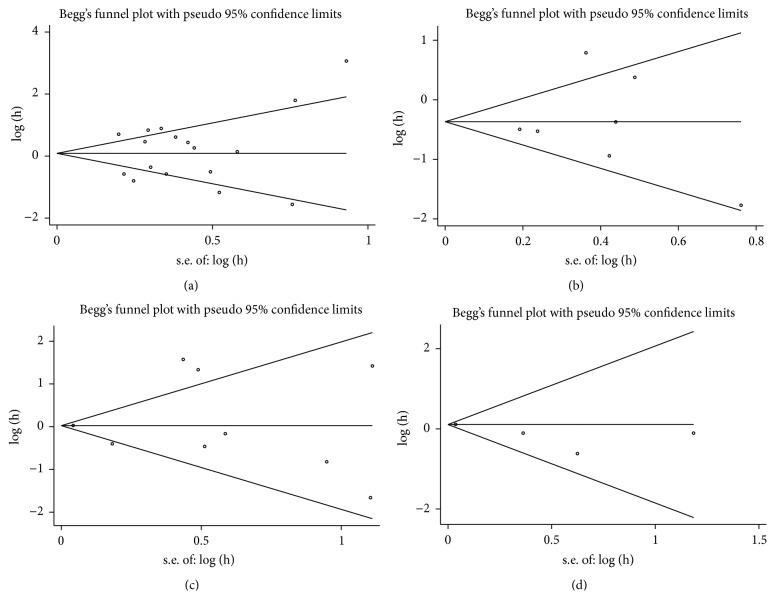
Begg's funnel plots of publication bias test. (a) OS associated with miR-200c; (b) DFS and PFS associated with miR-200c; (c) OS associated with miR-141; (d) DFS and PFS associated with miR-141.

Potential publication bias was assessed by Begg's funnel plot and Egger's test. Among 17 cohorts evaluating OS and 7 cohorts evaluating DFS/PFS for miR-200c, no obvious asymmetry was observed in Begg's funnel plots (Figures 6(a) and 6(b)), and the Egger's tests also showed no potential publication bias (OS: *t* = 0.45, *P* = 0.659; DFS/PFS: *t* = 0.09, *P* = 0.935). For miR-141, the funnel plots were symmetrical (Figures 6(c) and 6(d)) and the absence of significant publication bias was indicated by the *P* values of the Egger's tests among 9 cohorts evaluating OS (OS: *t* = 0.30, *P* = 0.771) and 4 cohorts evaluating DFS/PFS (DFS/PFS: *t* = −2.22, *P* = 0.156).

**Figure 7:**
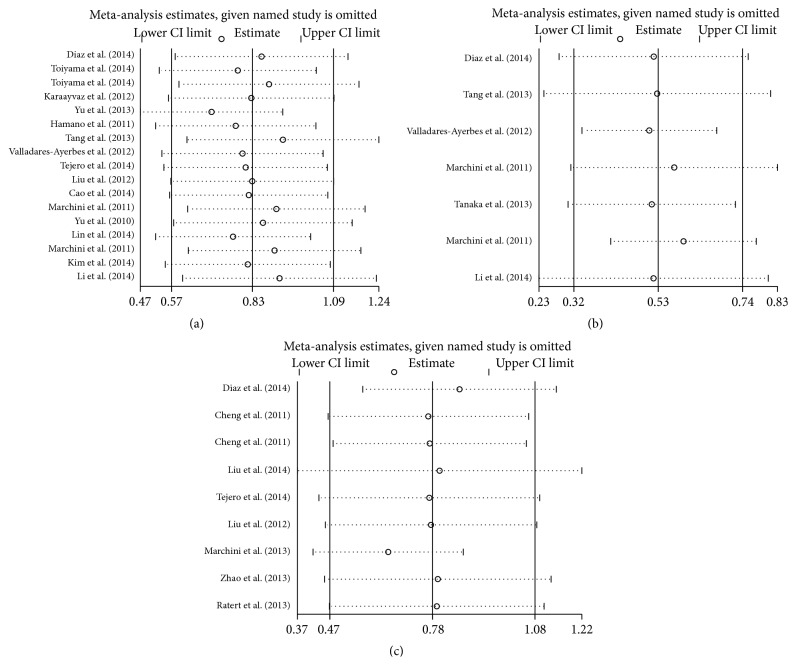
Sensitivity analysis. (a) effect of individual studies on the pooled HR for OS associated with miR-200c expression; (b) effect of individual studies on the pooled HR for DFS and PFS associated with miR-200c expression; (c) effect of individual studies on the pooled HR for OS associated with miR-141 expression.

The sensitivity analysis was performed by omitting each study at a given time to investigate the influence of any individual study on the stability of overall result. The results of sensitivity analyses for miR-200c with OS as the endpoint (Figure 7(a)) and DFS/PFS as the endpoint (Figure 7(b)) demonstrated that the pooled HRs were not significantly altered by removing every single study in sequence. Furthermore, in the sensitivity analysis for miR-141 with OS as the endpoint (Figure 7(c)), the result which was not changed confirmed the stability of the studies. Owing to the limitation of the number of eligible studies, the sensitivity analysis for miR-141 with DFS/PFS as the endpoint was not performed.

## 4. Discussion

Previous researches have indicated that miRNAs play important roles in tumorigenesis and cancer progression, which are closely related to many pathways such as innate and adaptive immune responses, cell cycle, angiogenesis, invasion, and metastasis [53]. Recent researches have revealed that acting as tumor suppressive or oncogenic genes, miRNAs exhibited a special expression profile in various cancerous tissues, which can be precisely detected and quantified by qRT-PCR in tissues and circulating samples, even in urine or saliva samples [10, 54]. Therefore, the miRNAs have been considered as novel potential biomarkers for cancer.

MiR-200c-141 genomic cluster, which located on chromosome 12p12.31, is the member of miR-200 family. Numerous researches have indicated that in different cancers the functional roles of miR-200 family members changed frequently, either as an oncogenic or as a tumor suppressive factor. Presumably, the expression of miR-200 family members may differ depending on the cellular contexts [31]. The role of miR-200c and miR-141 has been studied extensively in various cancers, but the conclusions are inconsistent.

As the first meta-analysis [55] of miR-200c related to outcomes of various cancers, Wang et al. retrieved 5 studies and found that lower level of miR-200c in tumor tissue and higher level of miR-200c in serum might be associated with worse overall survival in solid tumors. However, the obtained results might not be powerful, since the number of studies included was relatively small and the clinical outcome evaluated in this analysis was limited to OS. Particularly, hints to the specific settings for application of miR-200c as a prognostic biomarker might be missed because analyses of eligible studies were performed only according to sample types in this meta-analysis. In addition, although series of studies have explored the correlation between miR-141 and prognosis of various cancers, no meta-analysis has been published on this topic to summarize the evidence. In terms of this, we conducted the first comprehensive meta-analysis including 23 articles and showed that evaluated miR-200c expression cannot predict poor survival, local recurrence, and metastasis in patients with cancer. By stratified analyses of enrolled studies associated with miR-200c expression, we successfully drew some valuable conclusions.

First, in order to clarify the prognostic values of miR-200c in different source of samples, we classified the enrolled studies into subgroups of tissue samples and serum/plasma samples. We found that high level of miR-200c was significantly related to a poor OS in serum/plasma subgroup, but no statistical significance was defined in tissue subgroup for OS. Surprisingly but familiar to the results of previous meta-analysis by Wang et al. [55], our result demonstrated that elevated miR-200c expression can predict a significantly worse DFS/PFS in serum/plasma samples but a significantly favorable DFS/PFS in cancerous tissues. This may suggest that there was no direct correlation between circulating and matched tissue miR-200c expression.

Recent studies have revealed that the miR-200 family members exert important effects at distinct stages in tumor cell invasion and metastasis. The miR-200c have been indicated to regulate epithelial-to-mesenchymal transition (EMT) through the reciprocal miR-200-ZEB feedback loop, and the impaired expression of miR-200c induces EMT and promotes invasion and metastasis in various human tumors [14–18]. Across a diverse range of epithelial-derived cancer cell types, high miR-200c expression can enforce an epithelial state by repressing the expression of E-cadherin transcriptional repressors ZEB1 and ZEB2, whereas inhibited expression of miR-200c in mesenchymal cancer cells leads to upregulation of ZEBs and induces downregulation of E-cadherin. However, while miR-200 is downregulated in some cancers, upregulation of miR-200c has been found in multiple tumors indicating that miR-200c may also exhibit oncogenic potential, likely due to miR-200c overexpression increasing metastatic risk by the induction of MET. In the light of this, the prognostic value of miR-200c may vary in different cancers.

For this meta-analysis, the results indicated that high expression of miR-200c in circulation and low expression of miR-200c in tumor tissue were associated with worse survival in solid tumors. In the light of this finding, the expression of miRNAs in circulation and cancerous tissues cannot maintain consistency under some circumstance, which may be attributed to the hypothesis that the impact of miR-200c on the prognosis of cancer may be a process of dynamic change in tumorigenesis and tumor progression. The prognostic role of miR-200c may differ in distinct progression stages of tumor. In one of the included studies, Yuji Toiyama researched miR-200c expression in both serum and tissue and found that the matched metastases had higher expression level of miR-200c than the primary tumor. Accordingly, we speculate that low expression of miR-200c in tumor tissue may be related to a worse prognosis mainly in early stage of cancer. In such early stage, metastasis has not started yet, which is necessary to activate EMT as an initiating event of metastasis. Given the suppressive effect of miR-200c on EMT, tumors with upregulation of miR-200c have decreased the potential of invasion and metastasis by inhibiting EMT, ultimately leading to a favorable prognosis of cancer. With the increasing of tumor invasiveness, during the process of tumor metastasis, the positive prognostic role of upregulation of miR-200c in tissue may become less or even inexistent in advanced tumors and the negative prognostic value of high expression of circulating miR-200c may begin to raise in metastatic tumors. Therefore, circulating miR-200c was conjectured to be the origin of metastatic site.

Additionally, miR-200c plays a crucial role in regulating stem cell self-renewal and differentiation. This can be explained by the hypothesis that miRNA-200c in circulation may be a mirror of circulating tumor cells (CTCs). CTCs in peripheral blood can be a useful predictor of survival in various cancers [56]. It has been documented that the capacity of circulating miR-200c and miR-141 indicated the CTCs status, as well as its potential surrogate markers for CTCs and prognostic markers in patients with metastatic breast cancer [57]. This study also supports that circulating miR-200c in blood may be the origin of metastasis and overexpression of circulating miR-200c can be a valuable prognostic biomarker for advanced tumors.

Moreover, different secretory mechanisms and stability in blood and tissue may be the other reason accounting for the result. Besides, these conflicting results may be explained by different extraction and quantification methods. In current research associated with tumor prognosis, detection of miRNA in cancerous tissues has been widely used. However, compared to matched tissues, serum/plasma samples are easier and faster to access, and detection of miRNA in human peripheral blood has the advantages of low cost, convenience, and noninvasion. Particularly, because detection of circulating miR-200c is available at any time point during follow-up, it may be an efficacious method for dynamically monitoring the prognosis and evaluating recurrence risk for cancer patients.

Second, the enrolled studies associated with miR-200c were subgrouped into Asians and Caucasians according to ethnic affiliation in order to clarify the impact caused by the different genetic backgrounds on the results. Results indicated that high miR-200c expression was not a significant prognostic predictor for OS in both Asians and Caucasian populations. Interestingly, analyses revealed that high miR-200c expression was a significant favorable prediction for tumor progression in Asians, but not in Caucasians. Previous researches have demonstrated that specific miRNAs emerged diverse expression levels and predictive values in various ethnic groups [58–60]. These discrepancies may be attributed to the difference in hereditary backgrounds and environmental exposures. Finally, in order to further exclude the histological differences among various cancers, subgroup analysis was performed on the basis of cancer categories. We failed to find any statistical significance in subgroup analyses for OS, as well as in digestive system cancers subgroup analysis for DFS/PFS. However, it was observed that high level of miR-200cwas significantly associated with favorable DFS/PFS in urogenital and respiratory system cancers.

This meta-analysis indicated that high level of miR-141 did not predict cancer overall survival. Stratified analyses provide further confirmation, in both Asians and Caucasians, that no significant association was found between miR-141 expression and cancer overall survival. However, further analysis revealed that high level of miR-141 was correlated with a worse DFS/PFS. Based on the stratified analysis, we found that detected sample type had a considerable influence on the prognostic role of miR-141 expression. High level of miR-141 may be a significant predictor for poor survival and tumor progression in tissues but not in serum/plasma samples. Subgroup analysis on the basis of cancer categories revealed that in urogenital system cancers high level of miR-141 was suitable for predicting tumor progression.

What calls for special attention is that when interpreting the results of meta-analysis, heterogeneity is a potential and crucial issue that cannot be neglected [61]. In this meta-analysis, heterogeneity was observed in total comparison for overall survival on miR-200c and miR-141, as well as overall comparison for tumor progression on miR-200c. These results indicated that the pooled HRs of overall analyses are too crude to present accurate prognostic values of miR-200c and miR-141. Stratified analyses should be carried out to reduce the interference of heterogeneity. The heterogeneity was partly decreased in some subgroups when we conducted stratified analyses by classifying studies into subgroups of sample source, dominant ethnicity, and malignant diseases. However, heterogeneity still existed. Then sensitivity analyses were performed. We found that the estimated pooled hazard ratio changed quite a little when successively excluding each single study, which strengthened the results of this meta-analysis. The results suggested that ethnicity, sample, and cancer types may explain the heterogeneity observed in this meta-analysis. Moreover, lifestyle, environmental background, and other unknown aspects may also explain the results. No significant publication bias was shown implying these possible true results.

Admittedly, some limitations existed in this meta-analysis. Firstly, all literatures included in this meta-analysis were published in English although no restriction was set on retrieval. Concerns have been expressed on English language bias in meta-analytic researches [62, 63]. Secondly, the number of included studies was not sufficiently large for a comprehensive analysis despite the fact that no significant publication bias was detected in the meta-analysis. Furthermore, it is difficult to perform a meta-analysis in subgroups on the basis of the current finite sample size. This might weaken the reliability of meta-analysis results. Thirdly, several aspects which were not referred to in this meta-analysis might affect the pooled HRs. The heterogeneity was probably attributed to the differences in the patients characteristics, the clinical tumor stage, the cut-off criteria, the duration of follow-up, and so on. Owing to the absence of original information, data for survival were extracted from eligible studies based on univariate analysis without adjustment for age, gender, and other risk factors (e.g., dietary history and genetic predisposition to disease), which may cause confounding bias. These factors should be taken into consideration when drawing a conclusion. Finally, several HRs were calculated with the data estimated from survival curves, some minor differences exist between the exact HRs and the extrapolated data, according to Tierney's method [44].

In summary, we concluded that miR-200c and miR-141 expression in peripheral blood may be effective predictors for monitoring cancer progression and prognosis in the future. MiR-200c was suitable to predict tumor progression especially in Asians and urogenital system cancers and there was no direct correlation between peripheral blood and matched tissue miR-200c expression. Furthermore, High miR-141 expression was better at predicting tumor progression than patient survival for malignant tumors. To get a more comprehensive evaluation of the prognostic role of MicroRNA-200c-141 cluster expression in patients with cancer, more well-designed studies with larger sample sizes are needed.

## Figures and Tables

**Table 1:** Characteristics of the studies on miR-200c included in this meta-analysis.

Author	Publication year	Origin of population	Number of cases	Cancer type	Sample	Test method	Cut-off	Survival analysis	Estimated HR	Prognosis
Cao et al. [22]	2014	China	100	Ovarian cancer	Tissue	qRT-PCR	Median	OS	Reported	Negative

Lin et al. [23]	2014	Australia	97	Prostate cancer	Serum	qRT-PCR	Median	OS	Reported	Negative

Yu et al. [24]	2013	China	157	Esophageal cancer	Serum	qRT-PCR	Median	OS	Reported	Negative

Tanaka et al. [25]	2013	Japan	64	Esophageal cancer	Serum	qRT-PCR	Median	PFS	Reported	Negative

Diaz et al. [26]	2014	Spain	127	Colorectal cancer	Tissue	qRT-PCR	NA	OS; DFS	Estimated	Positive

Valladares-Ayerbes et al. [27]	2012	Spain	52	Gastric cancer	Blood	qRT-PCR	Mean	OS; PFS	Estimated	Negative

Marchini et al. [28]	2011	Italy	89; 55	Ovarian cancer	Tissue	qRT-PCR	25th quartiles	OS; PFS	Reported	Positive

Karaayvaz et al. [20]	2012	America	34	Endometrial cancer	Tissue	qRT-PCR	dCT = 35.5	OS	Reported	Positive

Toiyama et al. [29]	2014	Japan	156; 182	Colorectal cancer	Tissue; serum	qRT-PCR	ROC	OS; PFS	Reported	Positive; negative

Tang et al. [30]	2013	China	126	Gastric cancer	Tissue	ISH	An SI score of 2	OS; DFS	Estimated	Positive

Tejero et al. [31]	2014	Spain	155	Lung cancer	Tissue	qRT-PCR	NA	OS	Estimated	Negative

Liu et al. [32]	2012	China	70	Lung cancer	Tissue	qRT-PCR	2-fold	OS	Reported	Negative

Hamano et al. [33]	2011	Japan	98	Esophageal cancer	Tissue	qRT-PCR	Median	OS	Estimated	Negative

Yu et al. [34]	2010	Japan	99	Pancreatic cancer	Tissue	qRT-PCR	Value = 0.64	OS	Reported	Positive

Kim et al. [35]	2014	South Korea	72	Lung cancer	Tissue	qRT-PCR	Median	OS	Estimated	Positive

Li et al. [36]	2014	China	150	Lung cancer	Tissue	qRT-PCR	55th percentiles	OS; PFS	Reported	Positive

qRT-PCR: quantitative real-time polymerase chain reaction; ISH: in situ hybridization; NA: not available; ROC: receiver operating characteristic; SI (staining index score): combing staining intensity and proportion of positively stained cells; OS: overall survival; DFS: disease-free survival; PFS: progression-free survival.

**Table 2:** Characteristics of the studies on miR-141 included in this meta-analysis.

Author	Publication year	Origin of population	Number of cases	Cancer type	Sample	Test method	Cut-off	Survival analysis	Estimated HR	Prognosis
Cheng et al. [37]	2011	America; China	102; 156	Colorectal cancer	Plasma	qRT-PCR	Median	OS	Reported	Negative

Diaz et al. [26]	2014	Spain	56	Colorectal cancer	Tissue	qRT-PCR	NA	OS; PFS	Estimated	Positive

Liu et al. [38]	2014	China	212	Hepatocellular carcinoma	Tissue	ISH	An SI score of 3	OS	Reported	Positive

Tejero et al. [31]	2014	Spain	70; 72	Lung cancer	Tissue	qRT-PCR	NA	OS	Estimated	Negative

Selth et al. [39]	2013	Australia	70	Prostate cancer	Serum	qRT-PCR	Median	EFS	Reported	Positive

Liu et al. [32]	2012	China	70	Lung cancer	Tissue	qRT-PCR	2-fold	OS	Reported	Negative

Marchini et al. [40]	2013	Italy	52	Ovarian cancer	Tissue	qRT-PCR	25th quartiles	OS; PFS	Reported	Negative

Zhao et al. [41]	2013	China	40	Pancreatic cancer	Tissue	qRT-PCR	Median	OS	Estimated	Positive

Ratert et al. [19]	2013	Germany	40	Bladder cancer	Tissue	qRT-PCR	Median	OS	Estimated	Positive

Silva-Santos et al. [42]	2013	Portugal	90	Renal cancer	Tissue	qRT-PCR	Median	DSS	Estimated	Positive

qRT-PCR: quantitative real-time polymerase chain reaction; ISH: in situ hybridization; NA: not available; SI (staining index score): staining intensity × proportion of positively stained cells; OS: overall survival; DFS: disease-free survival; PFS: progression-free survival; DSS: disease-specific survival.
